# Nonpharmacological treatments for Tourette syndrome and tic disorders: A protocol for systematic review and network meta-analysis

**DOI:** 10.1097/MD.0000000000025741

**Published:** 2021-05-14

**Authors:** Hyo-Weon Suh, Chan-Young Kwon, Sunggyu Hong, Hyun Woo Lee, Misun Lee, Jong Woo Kim, Sun-Yong Chung

**Affiliations:** a College of Korean Medicine, Kyung Hee University, Dongdaemoon-gu, Seoul; b Department of Oriental Neuropsychiatry, College of Korean Medicine, Dong-eui University, Busanjin-gu, Busan; c Department of Clinical Korean Medicine, Graduate School, Kyung Hee University; d Kyung Hee University Industry Academic Cooperation Foundation, Dongdaemoon-gu; e Department of Neuropsychiatry, Kyung Hee University Korean Medicine Hospital at Gangdong, Gangdong-gu, Seoul, Republic of Korea.

**Keywords:** acupuncture, behavior therapy, biofeedback, deep brain stimulation, systematic review protocol, tic disorders, Tourette syndrome

## Abstract

**Background:**

A tic is a sudden, rapid, recurrent, nonrhythmic motor movement, or vocalization. Tic disorders are diagnosed based on the presence of motor or vocal tics, duration of tic symptoms, and age at onset. Current clinical practice guidelines strongly recommend behavioral therapies because they are more effective and safer than medications. To determine the most effective nonpharmacological intervention for tic disorders and Tourette syndrome, we will conduct a systematic review and network meta-analysis.

**Methods:**

We will search the Cochrane Central Register of Controlled Trials, MEDLINE, EMBASE, PsycARTICLES, AMED, 3 Chinese databases (China National Knowledge Infrastructure, Chongqing VIP, and Wanfang Data), 3 Korean databases (Korean Medical Database, Korean studies Information Service System, and ScienceON), and a Japanese database (CiNii). There will be no language or date restrictions. The primary outcome will be the tic severity scale, the Yale Global Tic Severity Scale. The secondary outcomes will include the effective rate defined by the trial authors, dropout rate, and adverse events. Methodological quality will be assessed using the Cochrane risk of bias tool.

**Results:**

Results of this review and network meta-analysis will be published in a peer-reviewed journal.

**Conclusions:**

This systematic review will assess the effectiveness of nonpharmacological interventions for treating tic disorders. A systematic review or meta-analysis will provide an unbiased overview of the existing evidence.

## 1 Introduction

A tic is a sudden, rapid, recurrent, nonrhythmic motor movement, or vocalization. Tic disorders are classified as Tourette syndrome (TS), persistent (chronic) motor or vocal tic disorders, and provisional tic disorders. TS is characterized by motor and vocal tics present for more than 1 year with childhood-onset.^[1]^ The pooled prevalence of TS is 0.52% (95% confidence interval [CI], 0.32–0.85) in children^[2]^ and 0.05% (95% CI 0.03–0.08) in adults.^[3]^ Although the exact mechanisms of tic disorders remain unclear, the following causes may be important:

1. dysfunction of the dopaminergic pathways within the cortico-striato-thalamo-cortical circuit,
2. deficits in cerebral maturation, and
3. environmental factors, such as infections, autoimmune dysfunction, or prenatal and perinatal problems.^[4]^

Current guidelines address antipsychotics, which regulate dopaminergic pathways, as pharmacological interventions for tics.^[5,6]^ The safety of antipsychotics has been investigated in schizophrenia; however, has not been well studied in tic disorders. It is unclear whether adverse events of antipsychotics (i.e., sedation, weight gain, cognitive impairment, and extrapyramidal symptoms) also occur at low doses in tic disorders.^[7]^

The American Academy of Neurology^[5]^ and European Society for the Study of Tourette Syndrome^[8]^ strongly recommend behavioral therapies because they are more effective and safer than medications. In addition, the American Academy of Neurology^[5]^ and European Society for the Study of Tourette Syndrome^[8,9]^ guidelines and a recent overview of systematic reviews^[10]^ recommended deep brain stimulation, biofeedback, and acupuncture as therapies for TS. These nonpharmacological interventions relieve tic symptoms through self-regulation practices (e.g., behavioral therapies and biofeedback) and external stimulation (e.g., deep brain stimulation and acupuncture).

Furthermore, information regarding the effectiveness of nonpharmacological interventions should be provided for shared-decision making among practitioners and patients/caregivers. Therefore, this review aims to confirm the effectiveness of nonpharmacological interventions by conducting further research. This information will be useful to health practitioners.

## 2 Objectives

To compare nonpharmacological interventions for tic disorders and TS, and to determine the most effective and safe nonpharmacological interventions.

## 3 Methods

This protocol will be conducted according to the Preferred Reporting Items for Systematic Reviews and Meta-Analyses Protocols statement^[11]^ and a checklist of items to include when reporting a systematic review involving a network meta-analysis.^[12]^ This research was registered on PROSPERO (CRD42021235476).

### 3.1 Inclusion criteria for study selection

#### 3.1.1 Types of participants

Children or adults with tic disorders, diagnosed by any validated criteria (e.g., Diagnostic and Statistical Manual of Mental Disorders, International Classification of Diseases, and Chinese Classification of Mental Disorders) will be included. We will record the diagnostic criteria for the tic disorders used in the included studies.

#### 3.1.2 Types of interventions

We will focus on the nonpharmacological interventions recommended by the European,^[6,8,9,13]^ Canadian,^[14]^ and American guidelines,^[5,15]^ and in a recent overview of systematic reviews.^[10]^ We will include both monotherapy and combination therapy as nonpharmacological interventions for tic disorders and TS. With respect to the combination therapy, combinations of only 2 interventions (e.g., any 2 of the nonpharmacological interventions or one of the nonpharmacological interventions plus one of the defined comparators) will be allowed for the intervention group. A combination of more than 2 interventions will be excluded. The selected nonpharmacological interventions are listed below:

##### 3.1.2.1 Behavior therapy and cognitive behavioral therapy

Inclusions: According to an earlier review,^[16]^ we will select behavioral therapies that have been recommended in the guidelines as follows: habit reversal therapy, comprehensive behavioral intervention, exposure and response prevention, cognitive behavioral therapy, contingency management and function-based interventions, relaxation training, self-monitoring, and awareness training.

Exclusions: Psychoanalysis, massed (negative) practices, and assertiveness training will be excluded. Supportive psychotherapy and psychoeducation will be excluded from the intervention, but will be included in the comparators.

##### 3.1.2.2 Deep brain stimulation

Inclusions: We will include all deep brain stimulations regardless of the location of the electrodes.

Exclusions: None

##### 3.1.2.3 Biofeedback

Inclusions: We will include all types of biofeedback training, including neurofeedback.

Exclusions: None

##### 3.1.2.4 Acupuncture

Inclusions: We will include acupuncture therapy using a penetrating needle (e.g., manual acupuncture, plum-blossom needle therapy, or intradermal need therapy). Additional stimulations to acupuncture, such as electroacupuncture and warm needling, will also be accepted. There will be no restrictions on whether needles are inserted at traditional acupoints. We will also include microsystem acupuncture, such as the ear (auricular), face, hand, scalp, and body acupuncture.

Exclusions: We will exclude non-penetrating acupuncture, such as laser acupuncture (photo acupuncture), acupoint magnet therapy, or acupressure. We will exclude acupuncture therapy that utilizes chemical stimulations, such as acupoint injections, bee venom therapy, or pharm acupuncture. Acupuncture therapy that does not use a needle, such as acupoint catgut embedding, will also be excluded.

#### 3.1.3 Types of comparators

We will include studies comparing different non-pharmacological interventions that meet the inclusion criteria. We will also include studies comparing nonpharmacological interventions with other active controls, including conventional medication, psychoeducation, watchful waiting, wait-list, no treatment, or sham control. The inclusion criteria for conventional medication according to the current clinical practice guidelines^[5]^ and a systematic review of antipsychotic drugs for tic disorders^[17]^ are as follows: typical antipsychotics (i.e., haloperidol, tiapride, pimozide, penfluridol, and fluphenazine), atypical antipsychotics (risperidone, olanzapine, aripiprazole, quetiapine, ziprasidone, paliperidone, sulpiride, and tetrabenazine), alpha-agonists, botulinum toxin injections, topiramate, or cannabis-based medications. However, we will exclude studies investigating different forms of specific interventions (e.g., manual acupuncture versus electroacupuncture).

#### 3.1.4 Types of outcome measures

##### 3.1.4.1 Primary outcome

The primary outcome will be tic severity, measured using validated scales. If the included study used various scales to measure tic severity symptoms, the Yale Global Tic Severity Scale^[18]^ will be used as the primary measurement, followed by the TS Clinical Global Impression Scale, Shapiro TS Severity Scale, Tourette Disorder Scale, Premonitory Urges for Tics Scale, or other scales. We will use these scales in this order based on previous systematic reviews and the authors’ opinions.^[17,19]^

##### 3.1.4.2 Secondary outcomes

Effective rate: An effective rate is the outcome measurement commonly used in trials conducted in China. The effective rate is the percentage of patients who responded to the intervention. Response levels (e.g., cure, effect, and no effect) were defined by the trial authors. We will adjust response levels that were defined differently in primary trials to dichotomous outcomes (e.g., effective or not). We selected a 25% reduction in the Yale Global Tic Severity Scale total tic severity score as the criterion value.^[20]^

Dropout rate: Compliance with treatment is an important outcome. Therefore, we will compare the dropout rates among the comparisons.

Adverse events: We will assess safety based on the number of adverse events in each group.

#### 3.1.5 Types of studies

Randomized controlled trials (RCTs) published in any language will be included. We will include parallel trials that assess the efficacy or effectiveness of nonpharmacological interventions as alternative monotherapies or adjunctive therapies for tic disorders. Crossover trials will also be included, but we will only use the data from before the crossover. Quasi-randomized trials will be excluded. If the method of random assignment is unclear, we will perform a sensitivity analysis and include trials that mention its limitations.

### 3.2 Search methods for identification of studies

The Cochrane Central Register of Controlled Trials, MEDLINE, EMBASE, PsycARTICLES, AMED, 3 Chinese databases (China National Knowledge Infrastructure, Chongqing VIP, and Wanfang Data), 3 Korean databases (Korean Medical Database, Korean studies Information Service System, ScienceON), and a Japanese database (CiNii) will be searched for RCTs. Our search strategy will examine patients (tic or Tourette) and interventions (behavior therapy, cognitive behavioral therapy, deep brain stimulation, biofeedback, or acupuncture) as the primary components. We will only use the RCT filter in the MEDLINE and EMBASE searches. There will be no language or date restrictions. Existing systematic reviews will be examined to identify additional trials. In addition, we will search the gray literature from the World Health Organization International Clinical Trials Registry Platform, United States National Institutes of Health Ongoing Trials Register (ClinicalTrials.gov), and Chinese Clinical Trial Registry.

The search strategies for the CENTRAL, MEDLINE, and EMBASE are presented in Appendix 1.

### 3.3 Data collection and analysis

#### 3.3.1 Selection of studies

Four review authors (HWS, SH, HWL, and ML) will independently screen the titles and abstracts for potentially relevant studies. After screening, the same 2 authors will independently assess the full-text articles for eligibility. Disagreements will be resolved through discussion. We will create a PRISMA flow chart with reasons for exclusion.^[21]^

#### 3.3.2 Data extraction and management

Four review authors (HWS, SH, HWL, and ML) will independently extract data using a standard data extraction form. We will extract data regarding the study design, setting, disease condition, diagnostic criteria, number of participants included, type of interventions and control, study duration and follow-up, outcome measures, and adverse events. Any disagreement will be resolved through discussion. If there are insufficient or missing data, we will contact the corresponding authors of the trials.

### 3.4 Quality assessment

Four review authors (HWS, SH, HWL, and ML) will independently assess the methodological quality using the Cochrane Collaboration tool for assessing the risk of bias.^[22]^ The following domains will be evaluated: random sequence generation, allocation concealment, blinding of participants and personnel, blinding of outcome assessments, incomplete outcome data, selective reporting, and other sources of bias. These 7 domains will be graded as having a low, unclear, or high risk of bias. Any disagreement will be resolved through discussion.

### 3.5 Data synthesis

#### 3.5.1 Pairwise meta-analysis

##### 3.5.1.1 Measures of treatment effect

We will present the outcomes as the mean difference with 95% CI for continuous data; if trials report outcomes with different scales, we will use the standard mean difference with 95% CI. With respect to dichotomous data, we will present the outcomes as relative risks with CI.

##### 3.5.1.2 Dealing with missing data

We will use an intention-to-treat analysis for the missing data. If data are missing or insufficient, we will contact the trial authors to obtain the missing data. A sensitivity analysis will be performed to assess the impact of missing data.

##### 3.5.1.3 Assessment of heterogeneity

Statistical heterogeneity will be assessed using the *I*^*2*^ statistic. According to the Cochrane Handbook, an *I*^*2*^ value >50% indicates substantial heterogeneity.^[22]^ If heterogeneity is observed, we will determine the potential sources of heterogeneity by conducting a subgroup analysis and sensitivity analysis.

##### 3.5.1.4 Assessment of reporting biases

Funnel plots can be used to assess reporting biases when a sufficient number of trials are included. However, there are different reasons for funnel plot asymmetry, including small-study effects or the presence of lower data quality and true heterogeneity in the included trials.^[22]^ If more than 10 trials are included, we will use funnel plots and conduct a sensitivity analysis to distinguish the reasons for funnel plot asymmetry.

##### 3.5.1.5 Data synthesis

Differences between the intervention and control groups will be assessed. We will conduct a meta-analysis using Review Manager software (version 5.3.5; Copenhagen: The Nordic Cochrane Center, Cochrane Collaboration. 2014). If *I*^*2*^ values are <50%, we will use a fixed-effects model. In the case of unexplained heterogeneity (*I*^*2*^ > 50%), a random-effects model will be applied.

#### 3.5.2 Network meta-analysis

If the included studies are of sufficient homogeneity and their clinical similarity and transitivity are judged acceptable, we will perform a network meta-analysis based on the frequentist method to estimate the comparative effectiveness of the included nonpharmacological interventions. A random-effects model will be applied for the analysis, and Stata/MP software version 16.0 (StataCorp LLC, TX) will be used. A network map will be created and presented to visualize the interrelationships between the interventions and number of studies included. A design-by-treatment interaction model will be used to detect inconsistencies between direct and indirect evidence, and the inconsistencies will be evaluated through the node-splitting method. In a network league table, the raw effect size data that will be calculated through a network meta-analysis will be presented. Furthermore, the best treatment will be identified by ranking it using the surface under the cumulative ranking curve. If a sufficient number of studies are included, a net funnel plot will be used to assess potential publication bias.

#### 3.5.3 Subgroup analysis

If the necessary data are available, subgroup analyses will be separately performed for high risk of bias (i.e., in sequence generation, allocation concealment, or incomplete outcome data), small sample sizes (less than 40 participants per group), and comorbidities (attention deficit hyperactivity disorder or obsessive-compulsive disorder).

### 3.6 Patients and public involvement

No patients or public will be directly involved in this review. Only the already existing data in the literature and aforementioned sources will to be used in this study.

## 4 Discussion

This systematic review will assess the effectiveness of nonpharmacological interventions for treating tic disorders. A systematic review or meta-analysis will provide an unbiased overview of the existing evidence.

### 4.1 Ethics and dissemination

This systematic review will not use data from individual patients, and ethical approval is not required. The results of this systematic review will be published in a peer-reviewed journal.

## Author contributions

**Conceptualization:** Hyo-Weon Suh, Chan-Young Kwon.

**Funding acquisition:** Sun-Yong Chung.

**Methodology:** Hyo-Weon Suh, Chan-Young Kwon, Sunggyu Hong, Hyun Woo Lee, Misun Lee.

**Writing – original draft:** Hyo-Weon Suh.

**Writing – review & editing:** Chan-Young Kwon, Jong Woo Kim, Sun-Yong Chung.

## Supplementary Material

Supplemental Digital Content
