# Supplementary material for: Nonpharmacological treatments for Tourette syndrome and tic disorders: A protocol for systematic review and network meta-analysis
Source: Medicine (Baltimore). 2021 May 14;100(19):e25741. doi: 10.1097/MD.0000000000025741 (PMC8133132; doi:10.1097/MD.0000000000025741)
Supplement: Supplemental Digital Content [file medi-100-e25741-s001.docx]

**1. CENTRAL**

| 1 | MeSH descriptor: [Tics] explode all trees |
| --- | --- |
| 2 | MeSH descriptor: [Tic Disorders] explode all trees |
| 3 | (tic?):ti,ab,kw |
| 4 | (habit* NEXT/1 (spasm* OR chorea*)):ti,ab,kw |
| 5 | MeSH descriptor: [Tourette Syndrome] explode all trees |
| 6 | (tourette?):ti,ab,kw |
| 7 | {OR #1-#6} |
| 8 | MeSH descriptor: [Psychotherapy] this term only |
| 9 | (psychotherap*):ti,ab,kw |
| 10 | MeSH descriptor: [Psychosocial Intervention] explode all trees |
| 11 | ((psychosocial NEXT/1 (intervention? OR therap* OR treatment?)) OR (psychological NEXT/1 (intervention? OR therap* OR treatment?))):ti,ab,kw |
| 12 | MeSH descriptor: [Behavior Therapy] explode all trees |
| 13 | (behavio* NEXT/1 (intervention? OR therap* OR treatment? OR modification? OR training)):ti,ab,kw |
| 14 | (conditioning NEXT/1 therap*):ti,ab,kw |
| 15 | (habit-reversal):ti,ab,kw |
| 16 | (habit-reversal):ti,ab,kw |
| 17 | ("awareness training" OR "competing response training" OR "relaxation training"):ti,ab,kw |
| 18 | (function* NEXT/2 intervention?):ti,ab,kw |
| 19 | ("response prevention"):ti,ab,kw |
| 20 | ("contingency management"):ti,ab,kw |
| 21 | MeSH descriptor: [Cognitive Behavioral Therapy] explode all trees |
| 22 | ((cognitive NEXT/1 (intervention? OR therap* OR treatment? OR training)) OR (cognition NEXT/1 therap*)):ti,ab,kw |
| 23 | (CBT OR CBIT):ti,ab,kw |
| 24 | MeSH descriptor: [Deep Brain Stimulation] explode all trees |
| 25 | ("deep brain" NEXT/1 stimulation?):ti,ab,kw |
| 26 | ((brain NEXT/1 (excitation OR stimulation? OR stimulus)) OR ("electrical brain" NEXT/1 stimulation?)):ti,ab,kw |
| 27 | MeSH descriptor: [Transcranial Magnetic Stimulation] explode all trees |
| 28 | ("transcranial magnetic" NEXT/1 stimulation?):ti,ab,kw |
| 29 | MeSH descriptor: [Biofeedback, Psychology] explode all trees |
| 30 | (biofeedback? OR neurofeedback? OR ((bio OR neuro) NEXT/1 feedback?)):ti,ab,kw |
| 31 | ("EEG feedback" OR myofeedback):ti,ab,kw |
| 32 | (neurotherap* OR (neuro NEXT/1 therap*)):ti,ab,kw |
| 33 | MeSH descriptor: [Acupuncture] explode all trees |
| 34 | MeSH descriptor: [Acupuncture Therapy] explode all trees |
| 35 | MeSH descriptor: [Acupuncture, Ear] explode all trees |
| 36 | MeSH descriptor: [Electroacupuncture] explode all trees |
| 37 | ("electrical acupoint stimulation"):ti,ab,kw |
| 38 | (acupuncture* OR electroacupuncture OR auriculoacupuncture OR acupoint* OR meridian*):ti,ab,kw |
| 39 | {OR #8-#38} |
| 40 | #7 AND #39 |

**2. MEDLINE/PubMed**

| 1 | Tics[MH] |
| --- | --- |
| 2 | "Tic Disorders"[MH] |
| 3 | tic[TIAB] OR tics[TIAB] |
| 4 | (habit*[TIAB] AND (spasm*[TIAB] OR chorea*[TIAB])) |
| 5 | Tourette Syndrome[MH] |
| 6 | tourette*[TIAB] |
| 7 | #1 OR #2 OR #3 OR #4 OR #5 OR #6 |
| 8 | Psychotherapy[Mesh:NoExp] |
| 9 | psychotherap*[TIAB] |
| 10 | Psychosocial Intervention[MH] |
| 11 | "psychosocial intervention*"[TIAB] OR "psychosocial therap*"[TIAB] OR "psychosocial treatment*"[TIAB] OR "psychological intervention*"[TIAB] OR "psychological therap*"[TIAB] OR "psychological treatment*"[TIAB] |
| 12 | Behavior Therapy[MH] |
| 13 | behavio*[TIAB] AND (intervention*[TIAB] OR therap*[TIAB] OR treatment*[TIAB] OR modification*[TIAB] OR training[TIAB]) |
| 14 | "conditioning therap*"[TIAB] |
| 15 | habit-reversal[TIAB] |
| 16 | self-monitoring[TIAB] |
| 17 | "awareness training"[TIAB] OR "competing response training"[TIAB] OR "relaxation training"[TIAB] |
| 18 | function* AND intervention*[TIAB] |
| 19 | "response prevention"[TIAB] |
| 20 | "contingency management*"[TIAB] |
| 21 | Cognitive Behavioral Therapy[MH] |
| 22 | "cognitive intervention*"[TIAB] OR "cognitive therap*"[TIAB] OR "cognitive treatment*"[TIAB] OR "cognitive training"[TIAB] OR "cognition therap*"[TIAB] |
| 23 | CBT[TIAB] OR CBIT[TIAB] |
| 24 | Deep Brain Stimulation[MH] |
| 25 | "deep brain stimulation*"[TIAB] |
| 26 | "brain excitation"[TIAB] OR "brain stimulation*"[TIAB] OR "brain stimulus"[TIAB] OR "electrical brain stimulation*"[TIAB] |
| 27 | Transcranial Magnetic Stimulation[MH] |
| 28 | "transcranial magnetic stimulation*"[TIAB] |
| 29 | Biofeedback, Psychology[MH] |
| 30 | biofeedback*[TIAB] OR "bio feedback*"[TIAB] OR neurofeedback*[TIAB] OR "neuro feedback*"[TIAB] |
| 31 | "EEG feedback"[TIAB] OR myofeedback[TIAB] |
| 32 | neurotherap*[TIAB] OR "neuro therap*"[TIAB] |
| 33 | Acupuncture[MH] |
| 34 | Acupuncture Therapy[MH] |
| 35 | acupuncture, ear[MH] |
| 36 | electroacupuncture[MH] |
| 37 | "electrical acupoint stimulation"[TIAB] |
| 38 | acupuncture*[TIAB] OR electroacupuncture[TIAB] OR auriculoacupuncture[TIAB] OR acupoint*[TIAB] OR meridian*[TIAB] |
| 39 | #8 OR #9 OR #10 OR #11 OR #12 OR #13 OR #14 OR #15 OR #16 OR #17 OR #18 OR #19 OR #20 OR #21 OR #22 OR #23 OR #24 OR #25 OR #26 OR #27 OR #28 OR #29 OR #30 OR #31 OR #32 OR #33 OR #34 OR #35 OR #36 OR #37 OR #38 |
| 40 | #7 AND #39 |
| 41 | "Meta-analysis as topic"[MH] OR "Meta-analysis"[PT] OR meta-analys*[TW] OR metaanalys*[TW] OR meta-synthes*[TW] OR metasynthes*[TW] OR "Systematic Reviews as Topic"[MH] OR "Systematic Review"[PT] OR (systematic-review*[TI] AND "Review"[PT]) OR ((systematic[TW] OR state-of-the-art[TW] OR scoping[TW] OR Integrative-literature[TW] OR Integrative[TW] OR rapid[TW] OR umbrella[TW]) AND (review*[TW] OR overview*[TW] OR assessment*[TW] OR literatur*[TW])) OR "research evidence"[TW] OR "Systematic"[sb] OR (("evidence-based medicine"[MH] OR evidence-based[TI] OR best-practice*[TI] OR evidence-synthes*[TW]) AND ("Review"[PT] OR "Behavior and Behavior Mechanisms"[MH] OR "Therapeutics"[MH] OR "Evaluation Study"[PT] OR "Validation Study"[PT] OR "Guideline"[PT])) OR "Consensus Development Conference"[PT] OR "Practice Guideline"[PT] OR ((cochrane[TIAB] OR EMBASE[TIAB] OR MEDLINE[TIAB] OR PubMed[TIAB] OR psychlit[TIAB] OR psyclit[TIAB] OR psychinfo[TIAB] OR psycinfo[TIAB] OR cinahl[TIAB] OR cinhal[TIAB] OR Science citation index[TIAB] OR Scopus[TIAB] OR Web of Science*[TIAB]) AND Database*[TIAB]) OR Reference-list*[TIAB] OR bibliograph*[TIAB] OR hand-search*[TIAB] OR relevant-journals[TIAB] OR manual-search*[TIAB] OR ((selection-criteria*[TIAB] OR inclusion-criteria*[TIAB] OR data-extraction*[TIAB] OR data-synthes*[TIAB]) AND "Review"[PT:NoExp]) OR systematic[sb] |
| 42 | "Randomized Controlled Trials as Topic"[MH] OR "Randomized Controlled Trial"[PT] OR RCTs[TW] OR RCT[TW] OR random*[TW] OR Controlled Clinical Trials as Topic[MH] OR "Controlled Clinical Trial"[PT] OR "controlled clinical trial*"[TW] OR "controlled trial*"[TW] OR "Clinical Trials as Topic"[MH] OR "Clinical Trial"[PT] OR "clinical trial*"[TW] OR "Clinical Trial, Phase III"[PT] OR "Clinical Trial, Phase IV"[PT] OR "Phase 3"[TW] OR phase3[TW] OR "phase III"[TW] OR P3[TW] OR PIII[TW] OR "Phase 4"[TW] OR phase4[TW] OR "phase IV"[TW] OR P4[TW] OR PIV[TW] OR "Multicenter Study"[PT] OR "Random Allocation"[MeSH] OR "Double-Blind Method"[MH] OR "Single-Blind Method"[MH] OR ((double*[TW] OR single*[TW] OR treb*[TW] OR tripl*[TW]) AND (blind*[TW] OR mask*[TW] OR dumm*[tw])) OR Placebos[MeSH] OR placebo*[TW] OR "Research Design"[Mesh:NoExp] |
| 43 | #41 OR #42 |
| 44 | #40 AND #43 |
| 45 | ((animals[Mesh] NOT humans[Mesh]) OR ("Comment"[PT] OR "Comment*"[TI] OR "Letter"[PT] OR "Letter*"[TI] OR "Editorial"[PT] OR "Editorial*"[TI])) |
| 46 | #44 NOT #45 |

**3. EMBASE**

| 1 | 'tic'/de |
| --- | --- |
| 2 | tic$:ab,ti |
| 3 | (habit* NEXT/1 (spasm* OR chorea*)):ab,ti |
| 4 | 'gilles de la tourette syndrome'/exp |
| 5 | tourette$:ab,ti |
| 6 | #1 OR #2 OR #3 OR #4 OR #5 |
| 7 | 'psychotherapy'/de |
| 8 | psychotherap*:ab,ti |
| 9 | 'psychosocial intervention'/exp |
| 10 | ((psychosocial NEXT/1 (intervention$ OR therap* OR treatment$)):ab,ti) OR ((psychological NEXT/1 (intervention$ OR therap* OR treatment$)):ab,ti) |
| 11 | 'behavior therapy'/exp |
| 12 | (behavio* NEXT/1 (intervention$ OR therap* OR treatment$ OR modification$ OR training)):ab,ti |
| 13 | (conditioning NEXT/1 therap*):ab,ti |
| 14 | 'habit reversal':ab,ti |
| 15 | 'self monitoring':ab,ti |
| 16 | ((awareness OR 'competing response' OR relaxation) NEXT/1 training):ab,ti |
| 17 | (function* NEXT/2 intervention$):ab,ti |
| 18 | 'response prevention':ab,ti |
| 19 | 'contingency management':ab,ti |
| 20 | 'cognitive behavioral therapy'/exp |
| 21 | ((cognitive NEXT/1 (intervention$ OR therap* OR treatment$ OR training)):ab,ti) OR 'cognition therap*':ab,ti |
| 22 | cbt:ab,ti OR cbit:ab,ti |
| 23 | 'brain depth stimulation'/exp |
| 24 | deep brain stimulation$":ab |
| 25 | ((brain NEXT/1 (excitation OR stimulation$ OR stimulus)):ab,ti) OR “electrical brain stimulation$”:ab,ti |
| 26 | 'transcranial magnetic stimulation'/exp |
| 27 | transcranial magnetic stimulation$":ab |
| 28 | 'biofeedback'/exp |
| 29 | biofeedback$:ab,ti OR bio feedback$":ab |
| 30 | 'eeg feedback':ab,ti OR myofeedback:ab,ti |
| 31 | neurotherap*:ab,ti OR 'neuro therap*':ab,ti |
| 32 | 'acupuncture'/exp |
| 33 | 'auricular acupuncture'/exp |
| 34 | 'electroacupuncture'/exp |
| 35 | 'electrical acupoint stimulation':ab,ti |
| 36 | acupuncture$:ab,ti OR electroacupuncture:ab,ti OR auriculoacupuncture:ab,ti OR acupoint*:ab,ti OR meridian*:ab,ti |
| 37 | #7 OR #8 OR #9 OR #10 OR #11 OR #12 OR #13 OR #14 OR #15 OR #16 OR #17 OR #18 OR #19 OR #20 OR #21 OR #22 OR #23 OR #24 OR #25 OR #26 OR #27 OR #28 OR #29 OR #30 OR #31 OR #32 OR #33 OR #34 OR #35 OR #36 |
| 38 | #6 AND #37 |
| 39 | 'meta analysis topic'/de OR 'meta analysis'/exp OR 'meta analys*':ab,ti,kw OR metaanalys*:ab,ti,kw OR 'meta synthes*':ab,ti,kw OR metasynthes*:ab,ti,kw OR 'systematic review (topic)'/de OR 'systematic review'/de OR ('systematic review$':ti AND 'review'/exp) OR ((systematic:ab,ti,kw OR 'state of the art':ab,ti,kw OR scoping:ab,ti,kw OR 'integrative literature':ab,ti,kw OR integrative:ab,ti,kw OR rapid:ab,ti,kw OR umbrella:ab,ti,kw) AND (review$:ab,ti,kw OR overview$:ab,ti,kw OR assessment$:ab,ti,kw OR literatur*:ab,ti,kw)) OR 'research evidence':ab,ti,kw OR (('evidence based medicine'/exp OR 'evidence based':ti OR ‘best practice$’:ti OR 'evidence synthes*':ab,ti,kw) AND ('review'/exp OR 'behavior'/exp OR 'therapy'/exp OR 'evaluation study'/exp OR 'validation study'/de OR 'practice guideline'/exp)) OR 'practice guideline'/exp OR 'consensus development'/de OR ((cochrane:ab,ti OR embase:ab,ti OR medline:ab,ti OR pubmed:ab,ti OR psychlit:ab,ti OR psyclit:ab,ti OR psychinfo:ab,ti OR psycinfo:ab,ti OR cinahl:ab,ti OR cinhal:ab,ti OR 'science citation index':ab,ti OR scopus:ab,ti OR 'web of science*':ab,ti) AND database*:ab,ti) OR ‘reference list$’:ab,ti OR bibliograph*:ab,ti OR 'hand search*':ab,ti OR 'relevant journals':ab,ti OR 'manual search*':ab,ti OR ((‘selection criteria$’:ab,ti OR ‘inclusion criteria$’:ab,ti OR ‘data extraction$’:ab,ti OR 'data synthes*':ab,ti) AND 'review'/exp) |
| 40 | 'randomized controlled trial topic'/de OR 'randomized controlled trial'/exp OR 'randomization'/exp OR rct:ab,ti,kw OR rcts:ab,ti,kw OR random*:ab,ti,kw OR 'controlled clinical trial (topic)'/exp OR 'controlled clinical trial'/de OR 'controlled clinical trial$':ab,ti,kw OR 'controlled trial$':ab,ti,kw OR 'clinical trial (topic)'/exp OR 'clinical trial'/exp OR 'clinical trial$':ab,ti,kw OR 'phase 3 clinical trial'/de OR 'phase 4 clinical trial'/de OR 'phase 3':ab,ti,kw OR phase3:ab,ti,kw OR 'phase iii':ab,ti,kw OR p3:ab,ti,kw OR piii:ab,ti,kw OR 'phase 4':ab,ti,kw OR phase4:ab,ti,kw OR 'phase iv':ab,ti,kw OR p4:ab,ti,kw OR piv:ab,ti,kw OR 'multicenter study'/de OR 'double blind procedure'/de OR 'single blind procedure'/de OR 'triple blind procedure'/de OR ((double$:ab,ti,kw OR single$:ab,ti,kw OR treble$:ab,ti,kw OR triple$:ab,ti,kw) AND (blind$:ab,ti,kw OR mask$:ab,ti,kw OR dumm$:ab,ti,kw)) OR 'placebo'/de OR placebo$:ab,ti,kw OR 'methodology'/de |
| 41 | #39 OR #40 |
| 42 | #38 AND #41 |
| 43 | animal'/exp NOT 'human'/exp OR comment*:ti OR 'letter'/de OR letter$:ti OR 'editorial'/de OR editorial$:ti OR 'abstract report'/de OR 'abstract report$':ti OR 'conference abstract'/de OR 'conference paper'/de OR 'conference review'/de OR 'data paper'/de OR 'erratum'/exp OR erratum:ti OR 'note'/de |
| 44 | #42 NOT #43 |
